# Society Reports

**Published:** 1903-04

**Authors:** 


### SOCIETY REPORTS.

### NEW YORK ACADEMY OF MEDICINE—SECTION ON ORTHOPEDIC SURGERY.

Meeting of November 21, 1902, George R. Elliott, M.D., Chairman.

Dr. Homer Gibney presented a cured case of Pott's disease in a child three years old, with the following history : Six months ago, (January, 1901) from no known cause he began to walk awkwardly ; the abdomen was thrown forward and he swayed from side to side with evident pain. Nothing was noticeable but the above till June, when a swelling appeared in the left buttock, which interfered with locomotion. He was treated at various hospitals and operated on at the Eighth Street Hospital by aspiration, pus and blood being withdrawn. Examination showed fairly well-nourished child; nodular swelling on left buttock posterior to the great trochanter, tense but not painful. Boy could not walk. The body was thrown forward, the abdomen prominent. In the effort to pick up a penny he supported himself by hands on knees. The lumbar spine was rigid and there was a slight prominence at the fourth lumbar vertebra. On August 1st a frame was applied and the grandmother was instructed in its care. December 20th, doing well, very little deformity; swelling on the left side had disappeared. January 11, 1902: Back in good condition, no deformity, fairly flexible. July 1, 1902: Has worn permanent jacket for two months, which was applied and allowed to dry on the frame, thus getting complete fixation in over-corrected position. November 18th : Left off the apparatus; spine flexible, no evidence of disease, discharged cured.

As to treatment, he said the frame was the one used in dispensary and private practice, a modification of the Bradford frame, made of canvas over gas-piping, easily bent from time to time, thus over-correcting the deformity.

*Pott's Disease—Three Cases under Treatment.*—Dr. Charlton Wallace presented three cases under treatment on the Whitman modification of the Bradford frame, the duration of the disease being twelve, eight, and six months respectively. He said, while as a rule cases over eighteen months of age are not treated by the frame, one of the cases presented was four years old and was doing well on the frame; in that case the disease had only lasted six months. The frame used is a parallelogram made of three-quarter inch gas-piping, four inches longer than the child, two inches excess at either end. It is made wide enough so that the side bars are opposite the glenoid cavities of the scapulæ. It is covered with tightly fitted canvas, laced in the rear. Two pads are arranged on each side of the focus of the disease, sewed to the canvas. Rubber cloth covers the canvas over the lower half for cleanliness. The apron is attached to three buckles on the sides by straps which are kept tight. The frame is gradually bent backward till the highest angle of the bend is at the site of the disease, so that the back is hyper-extended and the pressure relieved from the bodies of the vertebræ.

Dr. R. H. Sayre said he thought the treatment excellent. He used a similar treatment. He believed, however, that Dr. Wallace allowed the patient too much freedom of motion. He considered the motion of the legs unwise, as the play of the psoas muscle exercised a great deal of action on the spine, especially if the disease affected the lower dorsal or lumbar regions. In such cases the legs and trunk should be controlled; a jury-mast to the frame would be an advantage. He preferred the cuirass, allowing control of both extremities of the spine. It was also injurious to take the child off the frame for movements of the bowels and bladder, as so much moving would cause traumatism of the spine.

Dr. Royal Whitman stated that he had described and illustrated the treatment illustrated by the cases presented some years ago, and thought it better than the cuirass or other flat apparatus, because of the over-extension which this apparatus allowed. He further remarked that children were not removed from the frames for movements of the bowels, diapers or an ordinary dust-pan being used while the child was in position on the frame. Head and leg traction could be used if necessary, but that the position of over-extension itself was a great protection to the spine; it is also an advantage to have the side bars close together, as this allowed of less sagging than in the original Bradford apparatus. He further observed that the clothing adjusted over the frames was arranged to include the frame and the child. If further fixation were desired the child was suspended and a light plaster jacket applied. The patient was then replaced on the frame, to which its jacket must conform in hardening, thus assuring fixation and over-extension.

Dr. Wallace, replying to Dr. Sayre, said that none of the cases shown were lumbar, and a band was passed over the forehead to restrain head movements.

*Congenital Dislocation of the Hip.*—Dr. Whitman presented a patient operated upon five years ago for congenital dislocation of the hip, by the open method with enlargement of the acetabulum. But one side was operated upon as ankylosis appeared probable. Now the patient, a girl aged eleven years, limps on the unoperated side, and considers the ankylose limb as the "good one." The operated limb is two inches longer than the other and is much larger. There is no deformity, and practically no motion in the joint. This Dr. Whitman considered as bearing out his contention, advanced some years ago, that in the treatment of unilateral dislocation secure reposition—even if motion was very limited as the result of operative interference, provided there was no deformity, was a great improvement over unreduced displacement. He mentioned three similar cases to the one presented, and stated that he thought all of them had asked for operation on the other limb.

Dr. V. P. Gibney referred to a case similar to the one presented by Dr. Whitman, in which one side had been operated on, with ankylosis, the operated leg being the better. He spoke of another case, in which the operated limb was two and a half inches shorter and the thigh three inches smaller in circumference, yet the patient walked with ease. He noted the fact that patients and parents generally regarded these results from a different standpoint than the physician, and almost invariably begged for operation on the other leg.

Dr. S. A. Twinch asked Dr. Whitman how the patient would walk with both hips ankylosed.

Dr. Whitman stated that he had refused to operate on the second limb because he feared ankylosis. He did not present the case as showing a good result, but to demonstrate the contrast between the unoperated and the operated limbs, and further, that supposing this had been a unilateral dislocation the present result would have been far better than to have had a limb which would become progressively shorter.

Dr. George R. Elliott asked Dr. Whitman if he fixed an age limit for the open operation.

Dr. Whitman said he did not fix a positive age, but thought that the open operation could be done on older patients than in the case of the Lorenz operation.

Dr. L. W. Ely asked Dr. Whitman why he had not used the Lorenz operation on the other side.

Dr. Whitman thought it probable that the case has passed from observation; it was possible also that the Lorenz operation was not in such favor at that time.

*Noisy Shoulder.*—Dr. Sayre presented a patient, seen two months ago, giving the history of slight curvature of the spine accompanied by crackling of the muscles over the scapula on moving the shoulder up and down; there was also pain over the deltoid on the same side; the impression was given that the scapula was sliding over some substance. The case was presented for diagnosis and suggestions for treatment. Dr. Sayre referred to a somewhat similar case in an athlete who, after violently lifting weights, stated that he had pain along the erector spinæ muscles, with muscular cracklings.

Dr. V. P. Gibney said he had a similar case under observation in a young woman aged twenty, who had a noisy shoulder for a year. She had intercostal neuralgia and hysterical spine. He regarded the symptom as hysterical and prescribed paquelin cautery, with rest; marked improvement followed.

Dr. Whitman has seen several such cases and was impressed by the fact that the patients always wanted to produce the noise. He thought it was caused by a snapping tendon or possibly by a bursa beneath the scapula.

Dr. Homer Gibney stated that in giving exercises to patients he had noticed these crackling sounds in many cases, especially in one exercise for lateral curvature. He also thought it was a snapping tendon.

Dr. Elliot said he had seen similar cases and at present was treating a girl for lateral curvature of the spine who had the noisy shoulder to a marked degree. When he first saw her the scapula was quite immovable. She had been taking rather vigorous exercises under treatment, and as a result the scapula had became quite mobile and the noise was very marked. The slipping of tendons did not satisfactorily explain this objective symptom.

Dr. Leonard W. Ely read a paper entitled "A Case of Typhoid Spine." He referred to the summary and analysis of twenty-six cases reported by Dr. F. T. Lord in the Boston Medical and Surgical Journal of June 26, 1902, since Gibney reported his first cases in 1889, and said he had found three more cases reported.

The patient he referred to in his paper was a physician aged thirty-three years, who had a severe type of typhoid fever, January, 1902, complicated with pneumonia and pleurisy. At the end of six weeks, when the patient began to sit up, weakness of the back was observed. Following the convalescence in March, the weakness in his back continued and was made worse by an attempt to stoop. A lateral curvature and stiffness of the lumbar spine was observed, with some pain and great difficulty in standing erect. March 23d, a severe chill was followed by pneumonia of the left lung, complicated with pleurisy with effusion.

After three weeks, when the patient began to go about, the lateral curvature was quite marked and was accompanied as before by stiffness, weakness, and lumbar pain; no sensitiveness of spine to pressure. He went to Southern California early in May, but the spinal symptoms did not subside. The stiffness, weakness, and pain were increased by exertion. A severe cramp in the left lumbar region followed an attempt in rising from his chair, and returned each time he attempted to get up, so that he was compelled to keep his bed. When quiet in bed the pain did not return. A severe spasm in the left lumbar region, however, followed when turning over in bed. The spasm was tonic in character, lasting thirty seconds, gradually relaxing, then returned and was only relieved by chloroform inhalation after moderate use of morphine had failed. It was necessary to chloroform the patient when subjecting him to any effort.

The pain shifted to the right lumbar region, the spasms became less severe and were controlled by morphine. Examination showed patellar reflexes exaggerated, intermittent twitchings of the muscles of the thigh, no loss of sensation, no paralysis.

Spasms in the back continued, sometimes causing opisthotonos. Spasmodic attacks continued at intervals until July 27th. After this he often had the so-called "starting pain" upon falling to sleep. On September 22d a Taylor brace was applied and improvement from this on was rapid, but pain and stiffness in the right groin persisted for some time.

Stiffness and limitation of the lumbar spine were the only objective signs when he returned to New York, October 13th. He still wears the brace.

Referring to the pathology of typhoid spine, he said no autopsy had ever been reported. He believed it, however, an ostitis probably combined with a periostitis, and a probable neuritis caused by the inflammation of the bone and periosteum. This was generally considered to be the pathology of typhoid spine, but Osler thought it a neurosis and reported six cases.

Of the reported cases, twenty-six out of thirty were in males. The symptoms of the reported cases were much like those of lumbar Pott's disease, but more acute, and had the history of typhoid. Rest was the essential in treatment. Local applications did little or no good.

Dr. Whitman agreed with Dr. Ely that cases of typhoid spine were not so very uncommon. He had seen a number and had one case now under treatment at the hospital, a child ten years of age.

Dr. V. P. Gibney considered the history presented by Dr. Ely as the most complete on record, and agreed with him as to the frequency of the affection. There are all degrees of typhoid spine, some very mild and similar to those described by Osler. He had never been able to trace any definite trauma as cause. He was surprised that no relief was experienced from the cautery and thought it was not properly used, as he had obtained good results from it. He had nothing to add to the pathology. He thought the cases with kyphosis should not be included in the class of typhoid spines.

Dr. Whitman wished to know why cases were not to be included with typhoid spines in which kyphosis indicated destruction of the vertebræ.

Dr. Gibney answered that if the vertebræ were destroyed there would evidently be a destructive inflammation, which was apparently not the case with typhoid spines, the condition being rather one of overgrowth of tissue. He was inclined to think that in the deformed cases a tuberculous element was present.

Dr. Sayre referred to a case of what he called "diphtheritic spine" following an attack of diphtheria. The disease left a slight nephritis and spinal disturbance, attended with a certain amount of lateral deformity and interabdominal abscess formation, presumably the consequence of the diphtheritic infection.

Dr. Elliott asked Dr. Ely if in his research of the literature of typhoid spine he had found recorded marked relief following the application of a carefully applied support.

Dr. Ely replied that the effect was usually put down as being very quickly favorable. He regarded Dr. Gibney's remark about application of the cautery as a point well taken, and stated that the attending physician used the instrument at a black heat and made slow application—an operation the patient did not want to have repeated. He emphasized the necessity of rest in the treatment.

Dr. A. A. Berg presented a case of acute serous arthritis. An acute osteomyelitis of the femur had developed and the case was seen three days after the onset of the disease. There was considerable effusion in the knee joint. The medulla and lower epiphysis were opened and drained and the symptoms disappeared. There was no interference with function of the knee joint.

Dr. Berg read a paper entitled " The Joint Complications of Acute Pyogenic Osteomyelitis, with especial reference to the treatment of the Purulent Forms of Arthritis." He said : "The joint manifestations that may complicate acute pyogenic osteomyelitis are divided into the sympathetic or pseudo-arthritis and the true inflammatory arthritis. The former accompany the very early stages of the bone infection, and are often the first and only physical signs of such an acute osteomyelitis. This explains why these cases are so frequently diagnosed as acute articular rheumatism. The true arthritis is due to bacterial invasion of the affected joint. The sympathetic or pseudo-arthritis adds in no way to the severity of the septic symptoms arising from the primary bone infections, and tends to spontaneous subsidence after drainage of the marrow canal. The true bacterial arthritis adds materially to the patient's toxemia, itself requires surgical interference, and is apt to leave the joints seriously impaired in integrity and function."

The writer laid stress upon the periarthritis that accompanies the joint inflammation, whether the latter be of mild or severe form. It is of much importance diagnostically, inasmuch as the acute rheumatic and gouty forms of arthritis are at first confined to the joint serosa, with little or no periarthritis. The varieties of joint inflammation and their symptoms were briefly described.

The writer has minutely described his practice in the treatment of these joint complications. The suppurative types of arthritis he divides into two classes, in which a purulent exudate into the joint is the chief lesion, there being very little destructive change of the components of the joint, and into those in which the destructive inflammation of the ligaments, cartilages, and serosa is far more important than the purulent exudation into the joint. For the former, single or multiple incisions into the joint, with evacuation of exudate and drainage by tube or gauze, yield very good results ; in the latter he proposed that the suggestion of the Doctors Mayo, for such conditions of the knee joint (viz.: wide incision of the joint with partial dislocation of the bones, and dry gauze tamponade of the joint, with dressing in the dislocated position), be applied to the other large joints. He had employed this method in three very severe cases of suppurative goneitis, with excellent results ; and he felt assured that results equally satisfactory will follow the application to the other large joints. The technique of the operation for the knee and other joints was minutely described.

Dr. Sayre congratulated Dr. Berg on the result shown in the case presented. Regarding acute suppuration of the joint, he thought there was no question that radical operation in many cases would save amputation and give a useful joint.

Dr. V. P. Gibney spoke of the interesting array of surgical facts presented by Dr. Berg, and thought that the recognition of bone lesion by the earliest symptom, effusion into the joint, was a well-chosen point. In tuberculous arthritis of the knee, effusion into the joint is a well-recognized symptom before the bone symptoms appear. He thought these cases were often mistaken for rheumatic and traumatic synovitis.

Dr. R. H. Hibbs thought Dr. Berg's paper instructive, though his own experience in that line of work had been rather limited. He thought cases of synovitis supposed to be acute, but really manifestations of bone lesion, were common.

Dr. Elliott cited a case under his observation. The patient, a woman thirty-nine years old, had seven years ago run a needle into her finger. The acute symptoms readily subsided. There were no more symptoms for six months, at which time she became edematous and had pains in different parts of the body. The face, legs and arms became swollen and symptoms of general septicemia set in. The case was diagnosticated as neuritis in one of our large hospitals. The pain gradually subsided, with evidence of a multiple arthritis involving the elbows, knees, shoulders, ankles and hips. The difficulty lasted about eight months and subsided, leaving the knees ankylosed. In six months two popliteal abscesses formed and a quantity of pus was removed. When seen six months ago the legs were in contracture beyond a right angle. He had straightened the legs by brisement forcé. Dr. Elliott considered the interesting features of this case to be (1) that symptoms did not appear till six months after the trauma; (2) mistaking the case for one of neuritis, and (3) the faulty treatment in allowing the limbs to become contracted. The patient will in time be able to walk. He wished to ask Dr. Berg what was his opinion of the pathology of the joint lesion, as there was no pus in the joints and no known lesion in the ends of the bones.

Dr. Berg said he had seen such abscess formation after an osteoperiostitis. He stated that he was glad to learn that some chronic forms of joint lesion are attended with effusion in the early stages. He thought there was a great lack of knowledge among genera practitioners concerning joint diseases, as witnessed by the number of suppurative cases in the hospitals. He thought the type of sepsis after osteomyelitis was exceptionally rapid in its course.

---

### EDDYISM IN THE SIXTEENTH CENTURY.

"Until it is learned that generation rests on no sexual basis, let marriage continue." ("Science and Health, with Key to the Scriptures," one hundreth edition, 1896, p. 274.) If Mrs. Eddy had lived in the sixteenth century would she have indorsed the following decree of the Parliament of Grenoble, February 13, 1537, or would she, like most of us, have "winked the other eye"

Considering the evidence showing that it is more than four years since the said Lord of Aiguemère has carnally known the said Lady of Auvermont; considering the defense of the said lady, declaring that, although she has not carnally known her husband, yet having imagined in a dream the person and contact of the said Lord of Aiguemère, she experienced the same sensations of conception and pregnancy that she might have received in his presence, and affirming that, since the absence of her husband, for four years, she has hever had intercourse with any man, and that she has nevertheless conceived and borne the said Emmanuel, which she believes to have come about by the force of her imagination; considering the disposition of the Ladies of Albriche, of Pontrinel, of Orgeval, etc., affirming that such an accident may happen to women; that such things have happened to themselves and that they have conceived children of which they have been happily delivered, which resulted from certain imaginary intercourse with their absent husbands, and not from copulation; considering the attestation of the midwives and of the physicians; the court decrees that the said Emmanuel is and shall be declared the legitimate and true heir of the aforesaid Lord of Aiguemère, and charges the appellant to hold he said Lady of Auvermont as his wife in estate and honor.—*Gazette hebdomadaire des Sciences médicales de Bordeaux.*

### TREATMENT OF BRIGHT'S DISEASE.*

Permit me, as a basis for my remarks, to assume certain premises.

First. That all kidney lesions of Bright's are hematogenous, that is, the result of irritation produced by some specific kidney poison, as cantharides or the essential oils; or by the toxins of micro-organisms, as the Klebs-Lœffler bacillus; or by autoinfection, as from intestinal ptomains; or by the products of tissue waste, as uric acid.

Second. That broadly and simply considered, we meet three forms of the disease. (a) Acute diffuse nephritis, having a natural tendency toward recovery, yet also having a moderate death-rate and a slight probability of becoming chronic. (b) Chronic diffuse parenchymatous nephritis, large white or mottled kidney, capsule non-adherent, having a natural tendency toward death, yet, at least in comparatively early stages, capable of being arrested or even cured. (c) Chronic diffuse interstitial nephritis, cirrhotic kidney, adherent capsule, arteriosclerosis, cardiac complications, having a definitely fatal prognosis, yet amenable to treatment in the sense that its fatal termination may be held in abeyance almost indefinitely.

Too much stress cannot be laid upon hygienic therapeutics. It is not enough to give the patient a résumé of the hygienic principles involved. He must report at frequent intervals so that one may become familiar with his daily habit of life and assist him in regulating it from week to week for years.

### OCCUPATION.

Laboring men are especially prone to parenchymatous nephritis. Exposure to inclement weather, improper clothing and indulgence in alcohol account for the fact. Unfortunately, such patients have little choice of vocation and less opportunity to obey the laws of hygiene. Interstitial cases predominate among the well-to-do. The man who suffers from arteriorsclerosis should renounce active

---

*Read by John C. King, M.D., Banning, formerly President Southern California Medical Society, at Thirtieth Semi-Annual Session of the Southern California Medical Society, Pasadena, December, 1902.

business. The worry, the strain, the confinement, irregular hours and diet incident to competition must be stopped. The financial sacrifice involved is simply part of the cost of treatment.

CLIMATE.

Low altitude, equable temperature, moderate humidity, are desirable. The climate of the north Atlantic and lake coasts is unfavorable. Southern Georgia and Southern California are suitable to these cases, and those suffering from the interstitial form particularly, should be sent here promptly. Many cases are referred to me at Banning, but I am obliged to send them elsewhere. They do better nearer the coast. Our stimulating mountain climate is not well adapted to them. Some few do well at various points on the desert, but only where proper diet can be obtained.

CLOTHING.

The body must be well protected. Cotton permits too rapid evaporation from the surface. Authorities recommend woolen underwear all the year. If too irritating to the skin, cotton gauze may be worn underneath. I have found silk more valuable than any other material. It is expensive, but affords wonderful relief to gouty and rheumatic pain, and protects from the effect of sudden atmospheric changes. I believe that silk underwear is one of the very best prescriptions. Most of that on the market is part linen or even cotton, for economy's sake, but it will not answer. That made by the Ypsilanti Mills and sold in Los Angeles, at $14.00 per suit, has done me good service. It never shrinks. It is foolish to wear silk or wool during the day, then to sleep in a muslin gown. The animal texture should be worn constantly. In this climate a wrap should always be carried to protect from variation of temperature at sundown.

DIET.

In acute cases and in the occasional exacerbations in chronics, a milk diet is essential—a strictly milk diet. Rich milk, not skim milk. The patient must be nourished, not starved. I have never found one who could not live on milk, although many profess to be unable to take it, claiming it makes them bilious ( whatever that means). The addition of seltzer, vichy or lime-water may be required, or buttermilk or Koumyss may be substituted. Of course, an exclusive milk diet is not possible for years, nor is it desirable. I have found the Thomas diet lists, published by Saunders, to be extremely convenient and well adapted to the average case. Erasures or additions may be needed, to suit the individual. Pure water should be given in large quantities when the diet is not exclusively milk. Any simple bottled water will answer, aerated or not, according to patient's preference. Bethesda, Poland and Shasta are good examples. Both malt liquors and spirits should be absolutely prohibited. In interstitial cases a little dry wine may be useful. A feeble old man, with impaired digestion, hardened arteries and a failing heart may be benefited thereby.

### REST.

The acute case should be put to bed and kept there until convalescence ensues. This rule admits no exception. We have all had recoveries where the rule was violated, but the percentage is always against the patient. In chronics a certain amount of exercise is necessary to maintain nutrition, and yet, overexertion always increases the output of albumin. Instances where albumin is only present after exercise are sometimes observed. Mental rest is quite as needful as physical repose. It should not be forgotten that rest often means change of occupation rather than muscular inactivity. Patients undergoing the rest cure, no matter for what disease, should be subjected to expert massage.

### BATHS.

Daily sponging with tepid water will keep the skin in good condition, if followed by gentle but thorough friction. Both hot and cold baths are contraindicated. Even Baruch, the American apostle of the scientific use of water, cautions against baths in nephritis. Formerly the theory of rest for the kidney was in vogue and diaphoresis and purgation were practiced to obtain such rest. Later these methods were only used when uremia threatened or the patient became water logged. Steeping a man in water from fifteen to thirty minutes, at an ascending temperature of from ninety to 100 and then wrapping him in blankets will of course make him sweat. So will a hot air bath. But debility follows. The edema may be relieved, but it recurs, and the process is less satisfactory at each recurrence. In all chronic diseases the effort should be to build up, never to debilitate. In an emergency, in acute attacks, or when uremia threatens, such measures are sometimes of value. But even then, marked prostration, intense dyspnea and tendency to cardiac failure render their employment precarious. The hot pack, in blankets wrung from hot water, is, perhaps, less dangerous.

THE TEXT–BOOK ARTICLE

upon the treatment of Bright's consists, in the main, too frequently of a mere discussion of diuretics, diaphoretics and purgatives, as already stated; some advocate rest for the inflamed kidneys, and therefore oppose the use of diuretics. They would not treat enteritis by active purgation, nor gastritis by stimulating the flow of gastric juice, nor an inflamed kidney by increasing its functional activity. They obtain the desired rest by attempting to transfer the renal functions to the skin and bowels. The theory is beautiful. The results are inadequate. In acute cases, recovery usually follows any line of treatment, if combined with good hygiene—follows in spite of treatment. But the cardinal law in chronics is to build up, to support, to strengthen the patient. Continued diaphoresis and prolonged purgation do not conserve strength. Other therapeutists advocate diuresis. The kidney fails to act, the renal function is essential to life, Ergo: make it act; restore the function; very simple! Quite equal in simplicity to giving a purge for chronic constipation and quite as futile. Still others would seek the cause of the renal irritation and endeavor to remove it. If excess of uric acid, then limit its production by proper diet and neutralize it by alkalies. If autoinfection, attack the formation of ptomains in the intestine, and so on. This procedure is eminently rational. The difficulty is that causes are theoretical, are multiple and complicated, very difficult to isolate. Even when supposedly discovered, the problem of their removal remains unsolved. Another class of physicians does not attempt to theorize.

They point to the fact that when malarial fever was caused by a humor in the blood, quinine was an antiperiodic. Now, when malaria was produced by marsh miasm, quinine is the result of microorganism, quinine is an antiparaciticide. They depend upon the specific action of drugs, but do not pretend to analyze the theory of that action. If the truth be told, most of us practice empirical medicine. It obviates the necessity of knowing too much. When comparison is made between the results of all recognized methods of treating Bright's disease it will be found that the most successful plan is that which does the patient least harm. I do not wish to intimate that drugs have no place. In the presence of uremia we have all had brilliant results from heroic therapeutic measures too well known to admit of recapitulation in this short paper—we have seen convulsions and even coma pass away and the patient restored to a lease of life. We have seen the water-logged man reduced to normal proportions and his strength and usefulness return. Some twenty-eight years ago, in such a case, I prescribed the old-fashioned elaterium. Last year, in similar ones, I landed the new-fashion theobromine compounds, diuretin and agurin. The results were the same. Even more, we all believe, we have averted impending dropsy, or heart-failure, or uremia, by the timely administration of digitalis, or nitroglycerine, or some other pet remedy. We have seen gastric symptoms alleviated by hydrastine, nervous conditions by strychnine, and toxins carried off by calomel in minute doses. On the other hand, we have all seen the oma deepen into death and the heart fail in spite of our efforts, and some of us, at least, have had to face the half-formed suspicion that maybe we helped a little to hasten the end. I am unable to offer any therapeutic novelty. Two principals, however, I strive to adhere to religiously. First, good hygiene from beginning to end. Second, do the patient as little harm as possible.

Both the electro-therapeutist and the surgeon have recently added hronic Bright's to their respective specialties. It is quite easy for me to write upon this phase of the subject because I do not know anything about it, and I am, therefore, not embarrassed by the disagreeable consciousness of previous failure. Dr. Neiswanger, president of the Illinois School of Electro-Therapeutics, wrote to me, under date of November 7, that he considered the disease of central nervous origin; that he has been favored with signal success in all cases of parenchymatous and interstitial nephritis in which the heart has not developed the enlargement preceding mitral regurgitation. He refers to five physicians, names not given, who were treated six years, or more, ago, and who remain cured. The treatment is by static electricity and occupies from four to seven weeks. The technique is described in the 7th edition of his work on Electro-Therapeutical Practice. His fee for one month's treatment which, he claims, is usually sufficient to effect a cure, is $75. Dr. Champion, of Colton, assures me that he has observed a cure of one case of chronic interstitial nephritis under Neiswanger's care, and that he is familiar with the histories, including cures, of some eighteen or twenty cases reported by Dr. Neiswanger to the Cook county Medical Society. In all these cases the diagnosis and cure were said to have been verified by other physicians.

In the *Medical News* for April 22, 1899, Dr. George M. Edebohls, of New York, proposed to treat chronic Bright's by operation, and he further elaborated his proposition in the *Medical Record* for May 4, 1901. In the *Record* for December 2, 1901, he detailed eighteen cases. The diagnosis in these cases was verified by others and was based upon the clinical history, the chemical and microscopical examination of the urine, upon actual inspection and palpation of the kidney at the time of operation and in two cases upon microscopical examination cf bits of kidney tissue removed. One important pathological result of Edebohls's work is the apparent proof that chronic nephritis may be unilateral in nearly one-half the cases. If this hitherto unaccepted statement be assumed as fact, it will involve a radical change in our views. The operation consists of decapsulation of the kidneys with the object of creating new and liberal supplies of arterial blood to the diseased organ, through adhesions formed between the denuded kidney and the fatty capsule. In the *Medical Record* for September 3, 1902, Dr. J. A. Schmitt, of New York, controverts Dr. Edebohls's claims in a masterly manner and appends to his article a valuable bibliography.

A rehash of these papers would be tiresome. They are accessible to all. At any rate, the question must be settled by experiment, not by controversy. Upon November 1,

DR. EDEBOHLS WROTE

to me as follows: "I am continuing to perform decapsulation of the kidneys for chronic Bright's with encouraging success, haviug thus far operated upon forty-four patients. The result will be pubished in detail in due time." It may interest our local surgeons to know that, in a case of my own, a gentleman in ordinarily good circumstances, Dr. Edebohls fixed his fee at $1000. It is, therefore, evidently much better to treat these cases by operation than by electricity.

OUR OWN DR. LASHER, in a recent letter, assures me the problem is an experimental one; that the operation is easy; that he is prepared to perform all necessary experiments upon suitable cases. He closed with the encouraging remark that a few days before he had witnessed a death from the shock of the operation.—*Southern California Medical Journal.*

---

SENATOR STEWART of Ithaca, with an interest in the public health, quickened by the fact that his wife is ill with typhoid at his home, has introduced into the legislature a bill aiming at preventing the pollution of the waters of the State. The bill provides that permission will hereafter have to be obtained from the State Commissioner of Health for any person, shop, factory or industrial establishment to discharge sewage or other putrescible matter into the waters of the State, after the same shall have been forbidden by any local board of health or other public authority. The penalty for the discharge of sewage from any public sewer system without a permit is five hundred dollars, and fifty dollars a day while the nuisance continues. The penalty for discharging into any of the waters of the State any other matter prohibited by this bill is to be twenty-five dollars, and five dollars a day for each day the offense is maintianed. At the request of Dr. Frederick Holme Wiggin, President of the New York State Medical Association, the committee on legislation of that body is actively to support this bill.

### The King and His Doctors.

It is well known that the king occasionally suffers from his liver, and that the royal physicians are sometimes called in to treat him for his complaint. The latest occasion was the other day, when Sir Richard Powell, a prominent physician extraordinary to the king, was summoned to Buckingham Palace. The physician is a baronet of the old-fashioned school, with the pronounced characteristic of speaking his mind without regard to the social position of his patient. After asking his majesty a few questions in regard to his general health, the doctor laconically ordered him to strip. The king pleasantly asked what portion of his clothing he should take off. On being told to strip to the waist he quietly did so.

The doctor then proceeded to examine him in the usual manner, utilizing the stethoscope and another pencil-like instrument, until the king, who was not used to this vigorous sort of examination of all his organs, became anxious that it should conclude. Meanwhile Sir Francis Laking, physician to the king, entered and watched the procedure.

When the practical baronet bruskly pronounced: "You have eaten too much; you have drunk too much; I will send you a prescription to put you right," he departed with the scantiest ceremony. He had hardly reached the door when Sir Francis Laking, who was following him, overtook him and made a protest against his abruptness. The eminent specialist, who was apparently not in the best of humor, only retorted: "My dear Laking, if there is any squirming to do you must do it."

Dr. Laking returned to soothe his majesty's ruffled feelings, and remarked by way of palliation: "Sir Richard is a very busy man just now." The king's reply, which typified the state of his mind, was: "Good God, Laking, I thought he was going to tattoo me."

A story is told of the same physician, that when he was called to prescribe for the Duchess of Manchester, he ordered her to disrobe. "But, Sir Richard, I haven't my maid here," she said, to which the baronet retorted: "Madame, I have no intention of examining your maid."—*N. Y. Sun.*

